# Osteotomy and Osteoclasis for Deformities of the Lower Extremities

**Published:** 1885-01

**Authors:** 


**Osteotomy and Osteoclasis for Deformities of the Lower Extremities.** By CHARLES T. POORE, M. D., Surgeon to St. Mary's Free Hospital for Children, New York, etc. New York: D. Appleton & Co., 1, 3 and 5 Bond street. 1884.

There has been a want of a concise treatise on osteotomy, and the author aims in this work to supply the void. He has succeeded fully in his object, and presents to the profession, for their guidance in the treatment of a very common class of deformities, a very valuable work.

The contents are divided into ten chapters, as follows: The relations between rickets and certain deformities of the limbs; osteotomy; osteotomy for deformities at the hip-joint; genu valgum, its etiology and pathology; osteotomy for genu valgum; genu varum; osteotomy for anchylosis of the knee-joint; osteotomy for tibial curves; osteoclasis; statistics after osteotomies; bibliography. The subjects and diseases treated are such as the surgeon is compelled to consider and examine often in his daily practice, and the author has drawn from his own ample experience and that of other eminent surgeons in the preparation of a most admirable and useful work.

